# The role of mesenchymal stem cells in the occurrence, development, and therapy of hepatocellular carcinoma

**DOI:** 10.1002/cam4.4521

**Published:** 2022-01-03

**Authors:** Xiaoli Zhang, Na Li, Ying Zhu, Wei Wen

**Affiliations:** ^1^ Liver Disease Center of Integrated Traditional Chinese and Western Medicine The First Affiliated Hospital of Dalian Medical University Dalian Liaoning China; ^2^ Department of General Surgery The First Affiliated Hospital of Dalian Medical University Dalian Liaoning China

**Keywords:** combination therapy, hepatocellular carcinoma, liver cancer stem cells, mesenchymal stem cells, signaling pathway, tumor microenvironment

## Abstract

Hepatocellular carcinoma (HCC) is the most common type of liver malignant tumor, with high recurrence and mortality rates. Mesenchymal stem cells (MSCs) are multipotent cells that can be recruited into the tumor microenvironment (TME). What is known, TME plays a vital part in tumor progression. In recent years, accumulating studies have found that MSCs have a dual role of promotion and inhibition in the occurrence and development of HCC. In this review, we analyzed the role of MSCs in TME and summarized the relationship between MSCs and liver cancer stem cells, the molecular signaling pathway mechanisms of MSCs promoting and inhibiting HCC, and the latest research progress of MSCs in the treatment of HCC.

## Graphical abstract

The relationship between mesenchymal stem cells (MSCs) and hepatocellular carcinoma (HCC) is revealed comprehensively, including the molecular signaling pathway mechanisms of MSCs promoting and inhibiting HCC, the relationship between MSCs and liver cancer stem cells, and the latest research progress of MSCs in the treatment of HCC.

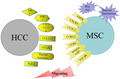

## 1 INTRODUCTION

Primary liver cancer is the seventh most common cancer and the second most common cause of cancer death in the world.^1^ Globally, hepatocellular carcinoma (HCC) is the main histological type of liver cancer, accounting for more than 75% of the total number of liver cancers.^2^ Liver cancer stem cells (LCSCs), recognized by certain surface markers, are responsible for the tumorigenesis, recurrence, metastasis, chemotherapy resistance and poor prognosis of HCC.^3^

Increasing evidence supports the great effects of the tumor microenvironment (TME) on the generation, development and metastasis of HCC. TME is composed of diversified cells and non‐cellular components.^4^ Mesenchymal stem cells (MSCs), an integral part of TME, are considered to be a key factor in tumor progression and metastasis. MSCs are also referred to as “mesenchymal stromal cells”, which can further be induced to differentiate into osteoblasts, chondrocytes, adipocytes, and other cells in vitro.^5^ Recently, massive research has been devoted to exploring the association between HCC and MSCs. The function of MSCs in the occurrence, development and treatment of HCC is quite controversial. A growing body of research illustrated that MSCs have the dual characteristics of suppressing and promoting tumors through different molecular signaling mechanisms. Even in different stages of HCC, MSCs also play a contradictory role. In the early stage of HCC, they can reduce DNA damage and ROS accumulation to play a tumor suppressor effect. However, in the late stage, MSCs manifested as a tumor promoter on HCC by promoting the stem cell‐like properties and epithelial‐mesenchymal transition (EMT).^6^

Abundant studies results in vitro or in vivo suggest that MSCs increase the number and tumorigenicity of CSCs in varied tumors, including human ovarian tumors,^7^ breast cancer,^8^ prostate cancer,^9^ Ewing's sarcoma,^10^ and colorectal Tumor,^11^ etc. Undoubtedly, HCC is no exception. In addition, the intrinsic ability of MSCs to treat HCC has been reported. Therefore, in this review, we stressed the role of MSCs in TME, the links between MSCs and LCSCs, the molecular signaling pathway mechanisms of MSCs acting on HCC (Table 1), and the current research status of MSCs in the treatment of HCC (Table 2).

**TABLE 1:** Mesenchymal stem cells (MSCs) promote or inhibit hepatocellular carcinoma (HCC) progression

Dual function	MSCs	Cell line	Impact on biological behavior	Mechanism	Reference
Promotion	hBMSCs	Bel7407, Huh7, LM3, Hep3B	Promote growth, migration and invasion	MAPK pathway, EMT, ITGA5	12
	hADMSCs	Hep3B, Huh7, HCCLM3	Inhibit proliferation, promote migration and invasion	ROS/MAPK/HIF‐1α signaling pathway	13
	hUCMSCs	7402, Hep3b	Promote growth	COX2/PGE2/EP4 axis, YAP, AKT/mTOR/SREBP1 pathway	14
	Canine ADMSCs	AZACH	Promote proliferation and invasion	TGFβ1, EGF, HGF, PDGFβ, VEGFA, IGF2	15
	HCC‐MSCs	293T, Hep3B, PLC, Huh7, HepG2, MHCC‐97L, HCC‐LM3, SMMC‐7721	Promote EMT and tumorigenesis	lncRNA‐MUF, ANXA2, miR‐34a, Wnt/β‐catenin	16
	hBMSCs	Bel‐7404, HepG2	Promote migration and invasion	IL‐6/STAT3 pathway	17
	BMSCs	SNU‐398	Promote migration and invasion	AQP1	18
	hBMSCs	SNU‐398	Promote migration and invasion	CXCR4	19
	UCMSCs	HCCLM3	Promote migration and invasion	TGF‐β	20
Inhibition	UCMSCs	H7402	Inhibit growth, migration and angiogenesis	—	21
	BMSCs, UCMSCs	HepG2	Inhibit growth and proliferation	—	22
	hUCMSCs	CCl4‐induced mouse liver tumor	Inhibit growth	anti‐oxidation	23
	UCMSCs	HepG2	Inhibit growth, promote apoptosis	AFP, Bcl‐2, Survivin	24
	hAMSCs	HepG2	Inhibit proliferation, promote apoptosis	Wnt/β‐catenin and IGF‐1R/PI3K/AKT pathway	25
	MenSCs	HepG2, HuH‐7	Inhibit growth	PI3K/AKT and MAPK pathway	26
	ADMSCs	HepG2/C3A/HB‐8065, PLC‐PRF‐5/CRL‐8024	Inhibit proliferation, migration and invasion, promote apoptosis	P53, retinoblastoma, c‐Myc, hTERT, TIMP‐1/2/3	27
Continued inhibition					
	Mouse BMSCs	The hepatic metastasis model of colorectal carcinoma	Suppress development, and promoted the anti‐tumor immunity	p65, iNOS, CD8^+^ T cells	28

**TABLE 2:** Mesenchymal stem cells (MSCs) combination therapy for hepatocellular carcinoma (HCC)

Combination therapy	Processing method	MSCs form	Mechanism	Effect	Reference
Combination chemotherapy	Sorafenib	MSCs	IL‐1, TNF–α, IL‐10	Promote apoptosis and inhibit proliferation	29, 30
	Adriamycin	MSC‐sFlt1	Anti‐angiogenesis	Prevent growth and induce apoptosis	31, 32
	Sorafenib	MSCs‐CM	—	Inhibit growth	33
	Adriamycin	ADMSC‐Exo‐199a	mTOR pathway	Improve chemotherapy sensitivity	34
	Sorafenib	MSC‐Exo‐siGRP78	Targeting GRP78	Inhibit growth and invasion, reverse drug resistance	35
Combination radiotherapy	Radiotherapy	AT‐MSCs	IFITM1, STAT3, MMPs, P53, P21, caspases	Inhibit growth, migration and invasion, and enhance the effect of RT treatment	36
	I131	SMAD‐NIS‐ MSCs	TGFB1	Delay growth and prolong survival	37, 38
Combination other therapy	Oncolytic adenovirus	MSCs	Extend virus cycle and improve safety	Enhance the efficacy of anti‐liver cancer	39
	rAd‐Apoptin	MSCs/MSCs‐CM	—	Inhibit proliferation	40
	Mel	MSCs	Induces apoptosis and inhibits inflammation and oxidative stress	Promote the therapeutic potential of MSCs	41
	Vitamin D	MSCs	Inhibit TGF‐β pathway	Improve pathological images, liver function, and promote the recovery of liver parenchyma	42
	GPC3‐ENG	MSCs	Activate T cells and produce IL2	Promote the killing of gpc3‐positive tumor cells	43
	rAd‐NK4	MSCs	Erk1/2	Inhibit growth and migration and tumor angiogenesis	44

## 2 MSCs IN THE TME

As a member of TME, MSCs have been reported in various tumor tissues such as pancreatic cancer, colon cancer, breast cancer, and gastric cancer.^45^, ^46^, ^47^, ^48^ A large number of studies have found that MSCs can also home to liver TME.^13^, ^16^ MSCs usually exist in a variety of human tissues, including bone marrow, adipose tissue, liver, intestine, lung, connective tissue, spleen, skin, placenta, umbilical cord and other tissues.^49^ Therefore, the homing property of MSCs makes the source of MSCs in TME diversified, including cells not only in liver situ but also recruited from a distance. Perhaps relatedly easier access, the current research on HCC‐related MSCs mainly obtained from bone marrow, fat, and umbilical cord.

**FIGURE 1:**
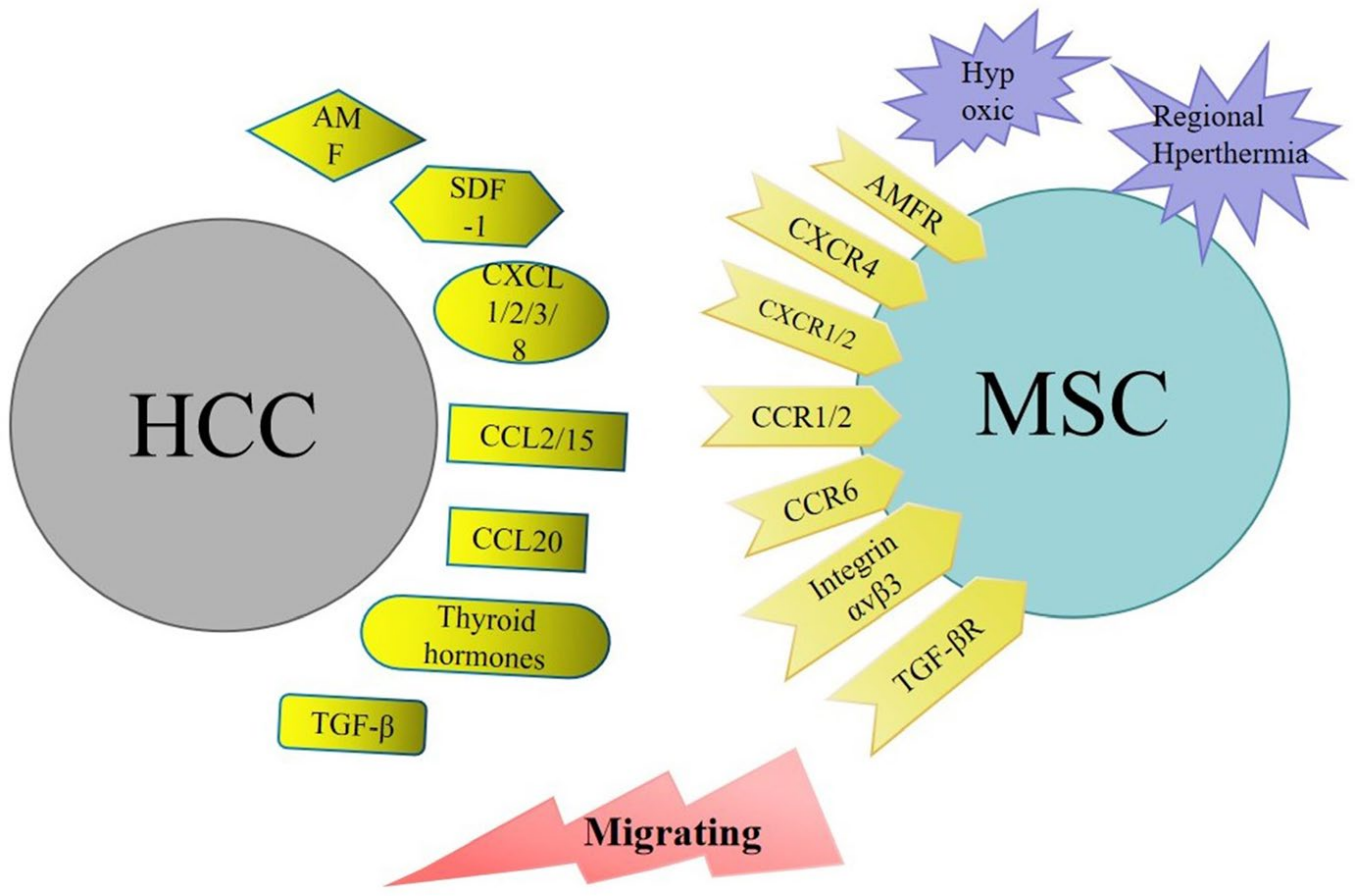
Chemotaxis mechanisms mediating mesenchymal stem cells (MSCs) migration to the hepatocellular carcinoma (HCC) microenvironment. MSCs. Chemokines in the TME (dark yellow) and external physical factors (purple) promote the migration of MSCs to the HCC microenvironment

The homing mechanisms of MSCs have been reported widely (Figure 1). Many soluble molecules secreted by hepatoma cells can induce MSCs migration toward HCC. Bayo et al. found that the migration of MSC to HCC is related to the chemotaxis axis of CXCL8/IL‐8, CXCL1‐2‐3/GRO, CCL2/MCP‐1 and AMF. Factors secreted by HCC regulate the chemotactic potential and gene profile of MSCs, thereby promoting their recruitment.^50^ CCL15, secreted by human HCC, has a chemotactic effect on hMSCs in vivo and in vitro via the CCL15/CCR1 axis.^51^ Lejmi et al. reported that MIP‐1δ and MIP‐3α are involved in the migration of pluripotent mesenchymal cells induced by liver cancer cells which may be related to the migration and evolution of MSCs to myofibroblasts around the tumor.^52^ CXCR4/ CXCL12 and TGF‐β/TGF‐βR signals also play an important role in the migration of MSCs to HepG2 cells.^53^ Interestingly, thyroid hormones can also increase hMSC migration to HCC stroma via integrin αvβ3.^54^ Interestingly, Chengying et al. found that HCC cells up‐regulated the expression of EGF, CXCL9, CCL25, and MMP‐9 in vivo and in vitro, which promoted the preferential transplantation of MSCs to the metastatic area rather than the primary tumor site.^55^ In addition, the induction of hypoxia and hyperthermia is also the reason for the enhancement of MSCs’ recruitment ability.^56^, ^57^ Although the phenomenon of pluripotent mesenchymal stem cells homing to tumors has been confirmed in succession, its tropism mechanism still needs to be further elucidated.

More and more evidence shows that MSCs can promote immune suppression by secreting varieties of cytokines in TME, such as IL‐10, TGFβ, nitric acid, indoleamine 2,3 dioxygenase and prostaglandin E2.^58^, ^59^ IFN‐γ and TNF‐α also play important roles in the immunosuppression of MSCs, for example, inhibiting the differentiation of dendritic cells (DC)^60^ and promoting the polarization of M2 macrophages,^61^, ^62^, ^63^ thereby promoting tumor growth.^64^, ^65^ The direct cell‐to‐cell interaction between MSCs and natural killer cells (NK) changes the phenotype and inhibits proliferation and cytokine secretion of NK cells.^66^ In addition, the soluble factors secreted by MSCs have been shown to inhibit the proliferation of T cells and b cells, while increasing the apoptosis of activated T cells.^67^, ^68^, ^69^ Meaningfully, Tumor secreted factors induce MSCs to differentiate into CAF phenotype through the TGF‐β/Smad signaling pathway.^70^, ^71^ CAFs inhibit lymphocyte tumor infiltration, increase the activity of immunosuppressive regulatory T cells, and induce monocyte apoptosis to promote immune tolerance to the tumor environment.^72^, ^73^, ^74^ In addition, it has been reported that CAFs damage the anti‐tumor function of T cells by activating neutrophils.^75^ A large amount of evidence indicates that MSCs can suppress the immune response to support tumor growth, whether it is induced by direct cell interaction or indirect differentiation.

Interestingly, MSCs also play a role in suppressing tumors by positively regulating the immune response. MSCs inhibit tumor growth by increasing the infiltration of monocytes and granulocytes in TME.^76^ In addition, bone marrow mesenchymal stem cells (BMSCs), which stimulate resting T cells and act as antigen‐presenting cells, activated by toll‐like receptor 3 (TLR3) enhance neutrophil function.^77^, ^78^ MSCs may also function in recruiting different immune groups into TME, changing the ratio of Treg and myeloid‐derived suppressor cells to CD8^+^ T cells, and shifting the balance to an anti‐tumor state.^79^ GPC3‐ENG MSCs redirected T cells to gpc3‐positive tumor cells and induced antigen‐dependent tumor cell killing, and MSCs overexpressing sirt1 also attracted CD8^+^ T cells approach.^28^, ^43^

### 2.1 The relationship between MSCs and LCSCs

CSCs could be isolated from tumor cell lines transformed from MSCs, speculating the possibility of CSCs origin from mutant MSCs.^80^, ^81^ The mutual transformation and influential mechanism of MSCs and CSCs have been reported in various tumors. For instance, the soluble mediator secreted by adipose‐derived mesenchymal stem cells (ADMSCs) advance cell proliferation and the phosphorylation of PI3K/Akt and MAPK signal‐related proteins in bladder cancer stem cell‐like cells.^82^ Lung adenocarcinoma stem cells are positively controlled by MSCs derived from Wharton´s Jelly (WJMSCs) through a paracrine mechanism.^83^

The relationship between MSCs and LCSCs has also been demonstrated. Studies have found that the phenotype and genotype of BMSCs are changed through HCC cells paracrine effects, generating a population of spherical stem cells with CSCs characteristics termed cancer‐induced stem cells (CiSCs). The Wnt/β‐catenin signaling pathway and TGF‐β may exert an enormous function on the transformation of BMSCs to CiSCs.^84^ SK cells are considered to be a human liver cancer cell line with properties of mesenchymal origin. Not only can they differentiate into osteoblasts and adipocytes like BMSCs and ADMSCs, but they show the relative homogeneity of CSCs, suggesting the feasibility of normal MSCs polarized into LCSCs with metastasis and self‐renewal capabilities.^85^ HCC cells turn into a more aggressive phenotype when co‐cultured with MSCs, which promotes HCC stemness and tumor metastasis.^16^ It has been reported that the lncRNA HULC and lncRNA MALAT‐1 play a certain part in both LCSCs and MSCs. LCSCs growth is boosted by both lncRNAs, and the synergistic effect of the two is more noticeable.^86^ MSCs overexpressed HULC represent stronger capabilities of proliferation, migration and invasion. Meaningfully, the proliferation, angiogenesis and immunosuppressive properties of MSCs are prominent by MALAT1 inducing VEGF and IDO.^87^, ^88^ Therefore, HULC and MALAT‐1 may be potential mechanisms in the interaction of MSCs and LCSCs. In addition, Hou et al. documented that irradiated MSCs (IR‐MSCs) can maintain the stemness of LCSCs through the Wnt/β‐catenin signaling pathway.^89^ In addition to HCC, exosomal miR‐126 derived from hepatoblastoma cells promotes the occurrence of hepatoblastoma by inducing BMSCs to differentiate into CSCs.^90^

## 3 MECHANISMS OF MSCs PROMOTING THE PROGRESSION OF HCC

### 3.1 The MAPK signaling pathway

The activation of the MAPK signaling pathway has been found in the progression of HCC. Human mesenchymal stem cells (hMSCs) promote the growth of HCC by activating the MAPK pathway to increase the expression of proliferation‐related proteins Ki‐67, pHH3, and PCNA, and boost the metastasis of HCC through EMT and ITGA5. Additionally, tissues treated with hMSCs showed a significant decrease of NK cell marker CD56 expression and an increase of TNF‐α and IL‐6 expression, which may contribute to tumor growth and metastasis.^12^ Of course, the effect between MSCs and HCC is mutually influential. When MSCs migrate to TME, MSCs are induced evolution into tumor‐associated mesenchymal stem cells (TA‐MSCs) by tumor cells through paracrine. After exposure to HCC conditioned medium (HCC‐CM), hADMSCs inhibit cell proliferation and enhance glycolysis by blocking the cell cycle and activating the mitochondrial apoptotic pathway, which can be reversed by withdrawing from HCC‐CM. Interestingly, the migration and invasion ability of hADMSCs is irreversibly enhanced under the action of HCC‐CM. On mechanism, changes in the phenotype and metabolism and permanent tumor‐promoting properties of hADMSCs are controlled by the ROS/MAPK/HIF‐1α signaling pathway.^13^

### 3.2 The Wnt/β‐catenin signaling pathway

Numerous research has been done on the transduction of the Wnt/β‐catenin signal as the pathogenic basis of the occurrence and development of HCC.^91^ The vital role of the Wnt/β‐catenin signaling pathway between MSCs and HCC also cannot be ignored. Moreover, various lncRNAs have been revealed to advance the progress of HCC. Yan et al. discovered a new type of lncRNA named lncRNA‐MUF, which is highly expressed in HCC tissues and strongly associated with poor prognosis. MSCs contribute to HCC's biological function through the interaction of lncRNA‐MUF with ANXA2 and miR‐34a. The Wnt/β‐catenin signaling pathway and EMT are activated by the bind between LncRNA‐MUF and ANXA2. In addition, lncRNA‐MUF, as a ceRNA competing with miR‐34a, leads to the up‐regulation of Snail1 and the activation of EMT, and ultimately promotes the malignancy of HCC.^16^

### 3.3 The IL‐6/STAT3 signaling pathway

The IL‐6/STAT3 signaling pathway is also involved in the beneficial behavior of MSCs to HCC. Large amounts of IL‐6 are secreted from BMSCs to stimulate IL‐6/STAT3 pathway signal transduction and significantly increase the invasion rate of liver cancer cells, which can be reversed by anti‐IL‐6 antibodies. Therefore, MSCs may strengthen the metastasis and invasion of HCC by activating the IL‐6/STAT3 signaling pathway.^17^

### 3.4 YAP related signaling pathways

Studies have found that the stimulation of YAP exerts a pivotal part in the effect of MSCs on HCC. Intriguingly, extensive literature indicated the active role of adipose tissue, adipose cells or ADMSCs in liver cancer progression. The progression of HCC can be induced by Hypoxic MSCs via activating YAP and regulating YAP‐mediated adipogenesis via the COX2/PGE2/EP4 axis. The mechanism is that hypoxia increases the expression of COX2 in MSCs and the secretion of PGE2, which then provokes YAP in HCC cells, leading to the proliferation of HCC cell lines and the growth of xenograft tumors. Simultaneously, adipogenesis in HCC cell lines is accelerated by YAP up‐regulating the AKT/mTOR/SREBP1 axis. Importantly, EP4 mediates the effect of a low concentration of MSCs on YAP activation and lipogenesis of HCC cells.^14^ Moreover, some phosphorylated kinases and NF‐κB signaling pathways, which are activated by exosomes from liver cancer cells, make adipocytes differentiated from MSCs have tumor‐promoting properties.^92^ Teshima et al. also elucidated the positive influence of AT‐MSCs soluble factors, such as TGFβ1, EGF, HGF, PDGFβ, VEGFA, IGF2, on the proliferation and invasion of canine hepatocellular carcinoma cells.^15^

### 3.5 Other molecular mechanisms

AQP1 is a known water channel that promotes metastasis and angiogenesis. Researchers found that the AQP1 level of liver cancer cells increased after exposure to BMSCs‐CM. In contrast, under the condition of AQP1 inhibitors, the migration and invasion of BMSCs‐mediated tumor cells were blocked, indicating that the HCC cell malignant behavior may be caused by the recruitment of BMSCs into the TME through AQP1 participation.^18^ Besides, CXCR4 also exerts a regulating role to hBMSCs and umbilical cord mesenchymal stem cells (UCMSCs) on HCC.^19^ 3D culture experiments have confirmed that TGF‐β participates in the metastasis and invasion progress of HCC advanced by UCMSCs that make no effect on liver cancer cell growth, drug resistance, and stem cell‐related gene expression.^20^ However, the reasons behind this apparent discrepancy need to be further investigated.

Most research is verified by conditioned medium or co‐cultivation experiments, so we should pay more attention to the interaction between MSCs and HCC. As researchers have discovered, HCC cells recruit MSCs and induce the phenotype of CA‐MSCs by expressing various cytokines that enhance migration. Liver cancer cells are empowered with excellent proliferation and migration by CA‐MSCs supernatant, demonstrating that MSCs may have a direct paracrine effect on tumor cells, thereby strengthening tumor angiogenesis, invasion and metastasis.

## 4 MECHANISMS OF MSCs INHIBITING THE PROGRESSION OF HCC

### 4.1 The anti‐tumor mechanism of UCMSCs

In recent years, the inhibitory effect of UCMSCs in the initiation and development of HCC has been demonstrated consecutively. Liu et al. described the inhibition effect of UCMSCs and UCMSCs‐CM on the growth, migration, metastasis and angiogenesis of HCC cells.^21^ The anti‐tumor characteristic of BMSCs and UCMSCs to HCC, especially UCMSCs, is also pointed out by Alshareeda et al.^22^ Mechanism studies disclosed that exosomes derived from hUCMSCs (hucMSC‐Ex) reduce the oxidative stress level of liver tumors, thereby suppressing the acute liver injury and fibrosis induced by CCl4 and the growth of liver tumors, and significantly reducing tumor size and inflammation infiltration area.^23^ Tang et al. found that UCMSCs may restrain the growth of co‐cultured hepatocarcinoma cells by down‐regulating AFP, Bcl‐2 and Survivin, and accelerate cell apoptosis which is related to the apoptosis signal pathway. Furthermore, UCMSCs function in a time‐dependent and cell‐number‐dependent manner.^24^

### 4.2 The anti‐tumor mechanism of ADMSCs

Interestingly, numerous previous literature reported the promotion function of ADMSCs on liver cancer. However, Serhal et al. investigation highlighted its tumor suppressor effect on HCC. When HCC cells are co‐cultured with ADMSCs or treated with ADMSCs‐CM, the apoptosis rate of HCC increases and the proliferation is significantly hindered, which is accompanied by the up‐regulation of P53 and retinoblastoma mRNA, as well as the down‐regulation of c‐Myc and hTERT mRNA levels. Notably, ADMSCs and ADMSCs ‐CM inhibit the expression of two important HCC carcinogenic markers‐ AFP and DCP. Furthermore, the level of migration and invasion of liver cancer cells was remarkably reduced, which may be due to the increased expression of TIMP‐1/2/3.^27^

### 4.3 Tumor suppressor signaling pathways of other types of MSCs

In addition, varieties of signaling pathways are involved in the process of MSCs inhibiting HCC. Previous literature showed that MSCs could regulate HCC proliferation negatively through NF‐κB, Notch1, Akt, and TGF‐β signaling pathways.^93^, ^94^, ^95^, ^96^ Currently, other signaling pathways have also been discovered consecutively. Human amniotic mesenchymal stem cells (hAMSCs) have notable anti‐tumor effects both in vivo and in vitro. hAMSCs paracrine a variety of cytokines, such as DKK‐3, DKK‐1 and IGFBP‐3, which suppress the proliferation and promote the apoptosis of liver cancer cells by blocking the Wnt/β‐catenin and IGF‐1R/PI3K/AKT signaling pathways.^25^ Factors released by MSCs, such as DKK‐1, compete with Wnt for binding to LRP5/6, thereby inhibiting the Wnt signaling pathway.^97^, ^98^ The abundance and distribution of 5‐hmC and 5‐mC in the regulatory region are regulated by Human menstrual blood‐derived stem cells (MenSCs) to silence PI3K/AKT and MAPK carcinogenic pathways and chemotherapy resistance‐related genes including ID4 and HMGA1 and activate tumor suppressors. The inactivation of the MAPK pathway further destroys the c‐Myc‐mediated EMT. Briefly, HCC growth is controlled by MenSCs, especially function in enhancers and promoters.^26^ The tumor suppressor effect of some genes on MSCs has also been confirmed. For example, MSCs overexpressing the pro‐inflammatory regulator sirt1 secrete chemokines to repress iNOS in an inflammatory environment, which further attract CD8^+^ T cells to approach without inhibiting their proliferation. Ultimately, the anti‐tumor effect is driven by its pro‐inflammatory ability.^28^

## 5 THE MSCs‐BASED HCC THERAPIES

Accumulating studies with in vitro experiments and animal models have shown the ability of MSCs homing to TME. The migration capacity of MSCs is raised by highly tumorigenic HCC cells, which makes MSCs an ideal carrier for targeted therapy of liver cancer.^99^ Compared with anti‐tumor monotherapy, the superiorities of synergistic combination therapy are including promoting the efficiency and specificity of MSCs‐mediated anti‐cancer, reversing drug resistance, improving radiotherapy potential and safety.

### 5.1 The combination therapy of MSCs and sorafenib

Contrasted to sorafenib alone, sorafenib combined with MSCs in the treatment of liver cancer gives rise to HCC cell apoptosis and attenuates tumor cell proliferation. Mechanism studies have discovered that MSCs reduced and increased the concentrations of tumor‐promoting factors such as IL‐1 and TNF‐α, and tumor suppressor IL‐10 respectively. Conclusionally, the anti‐tumor and anti‐metastatic features of MSCs are retained by combined with sorafenib.^29^, ^30^ The inhibitory effect of MSCs‐CM on the growth of liver cancer cells has been reported in several pieces of literature. Seyhoun et al. further found that sorafenib combined with MSCs‐CM also has a negative result on the growth of liver cancer cells. Meaningfully, the synergistic effect of sorafenib and 80% MSCs‐CM in HCC cells is of clinical significance, allowing the use of lower doses of sorafenib and being more effective than currently single use.^33^ More precise drug‐resistant targets have already arisen. For example, GRP78, overexpressed in sorafenib‐resistant liver cancer cells, is used as a therapeutic target of siGRP78‐modified MSCs exosomes combined with sorafenib in HCC cells, thereby weakening the growth and invasion of cancer cells in vivo and in vitro, reversing sorafenib resistance.^35^

### 5.2 The combination therapy of MSCs and adriamycin

sFlt1 is a promising VEGF inhibitor, which is becoming a new method to destroy angiogenesis. After genetic engineering, MSCs that secrete sFlt1 possess a marked anti‐angiogenesis effect in HCC, thereby hindering tumor growth. The growth of liver cancer and cell apoptosis can be prevented and induced separately by combined treatment of sFlt1 genetically engineered MSCs and continuous low‐dose adriamycin. Importantly, the effectiveness and safety of this combination therapy are verified through in vivo experiments.^31^, ^32^ MiR‐199a‐3p, the third most highly expressed miRNA in the normal liver, is down‐regulated in almost all HCC cells and associated with poor prognosis. And mTOR has been identified as the direct target of miR‐199a‐3p. Besides, exosomes derived from MSCs have tumor suppressor effects.^100^ Hence, Lou et al. observed the adriamycin sensitivity of HCC was reinforced by mir‐199a modified adipose tissue‐derived mesenchymal stem cell exosomes (ADMSC‐Exo‐199a) through the mTOR pathway.^34^

### 5.3 The combination therapy of MSCs and radiotherapy

ADMSCs can enhance the potential of radiotherapy (RT) for liver cancer. ADMSCs significantly destroy the ability of liver tumors growth, migration and invasion, and enhances the tumor regression effect of RT therapy by inhibiting IFITM1 gene expression, which mechanism is attributed to the down‐regulation of STAT3 and MMPs and the up‐regulation of P53 and caspases.^36^ The effectiveness of the combination treatment of NIS genetically modified MSCs and I131 in HCC has also been confirmed. SMAD‐NIS‐MSCs have a high recruitment rate in the tumor stroma. And the SMAD promoter activity induced by TGFB1 takes with a strong biological targeted NIS transgene expression in subcutaneous HuH7 tumors. The noticeable therapeutic effects, including a delay in tumor growth and prolonged survival, are gained by the systemic application of SMAD‐NIS‐MSCs followed by I131 injection. This remarkable therapeutic effect is believed to be largely related to TGFB1 which leads to highly selective and focused amplification of MSCs‐based NIS expression in the tumor environment.^37^, ^38^

### 5.4 Therapy of genetic modified MSCs

Apoptin from the chicken anemia virus is a protein with an inherent ability to dissolve cancer cells without harming normal cells. By modifying the adenovirus (Ad) vector, therapeutic gene expression with low toxicity and high transferability can be achieved. The proliferation of liver cancer cells can be significantly restrained by both apoptin‐modified MSCs and CM in a dose‐dependent manner.^40^ In addition, investigators described T cells were redirected to GPC3^+^ tumor cells by GPC3‐ENG MSCs, which may become a GPC3‐targeted treatment method. GPC3‐ENG MSCs express CD80 and 41BBL, which activate human T cells to produce IL2 in an antigen‐dependent manner and facilitate the proliferation of T cells, thereby killing the GPC3^+^ tumor cells.^43^ NK4 can not only limit the growth, metastasis and invasion of tumor cells induced by HGF, but suppress tumor angiogenesis independent of the HGF/c‐Met pathway. MSCs modified by rAd‐NK4 apply negative effects on the growth and migration of liver cancer cells and tumor angiogenesis, which are associated with the inhibition of Erk1/2 phosphorylation, providing a new strategy for HCC targeted therapy.^44^ HMGA1 and BYSL have also been found to be potential targets of genetically modified MSCs for liver cancer treatment.^26^

### 5.5 Other combination therapies

MSCs‐mediated systemic delivery of oncolytic adenovirus (oAd) is a promising strategy that can strengthen the efficacy of anti‐liver cancer and improve safety at the same time. The virus replication in the MSCs vector is active. oAd accumulates specifically due to the homing of MSCs to tumors, and because of the cargo‐protective of MSCs, the circulation of virus particles in the blood is extended. Safety is improved by reducing liver sequestration and liver toxicity, and the elimination of MSCs is advanced through viral replication.^39^ El‐Magd et al. have elucidated that Melatonin (Mel) maximizes the survival and therapeutic potential of MSCs in HCC possibly by inducing cell apoptosis and eliminating inflammation and oxidative stress.^41^ In chemically induced HCC rats, the combination treatment of MSCs and vitamin D improves liver function and the recovery of liver parenchyma with better pathological images by hindering the TGF‐β signaling pathway.^42^

## 6 CONCLUSION

**FIGURE 2:**
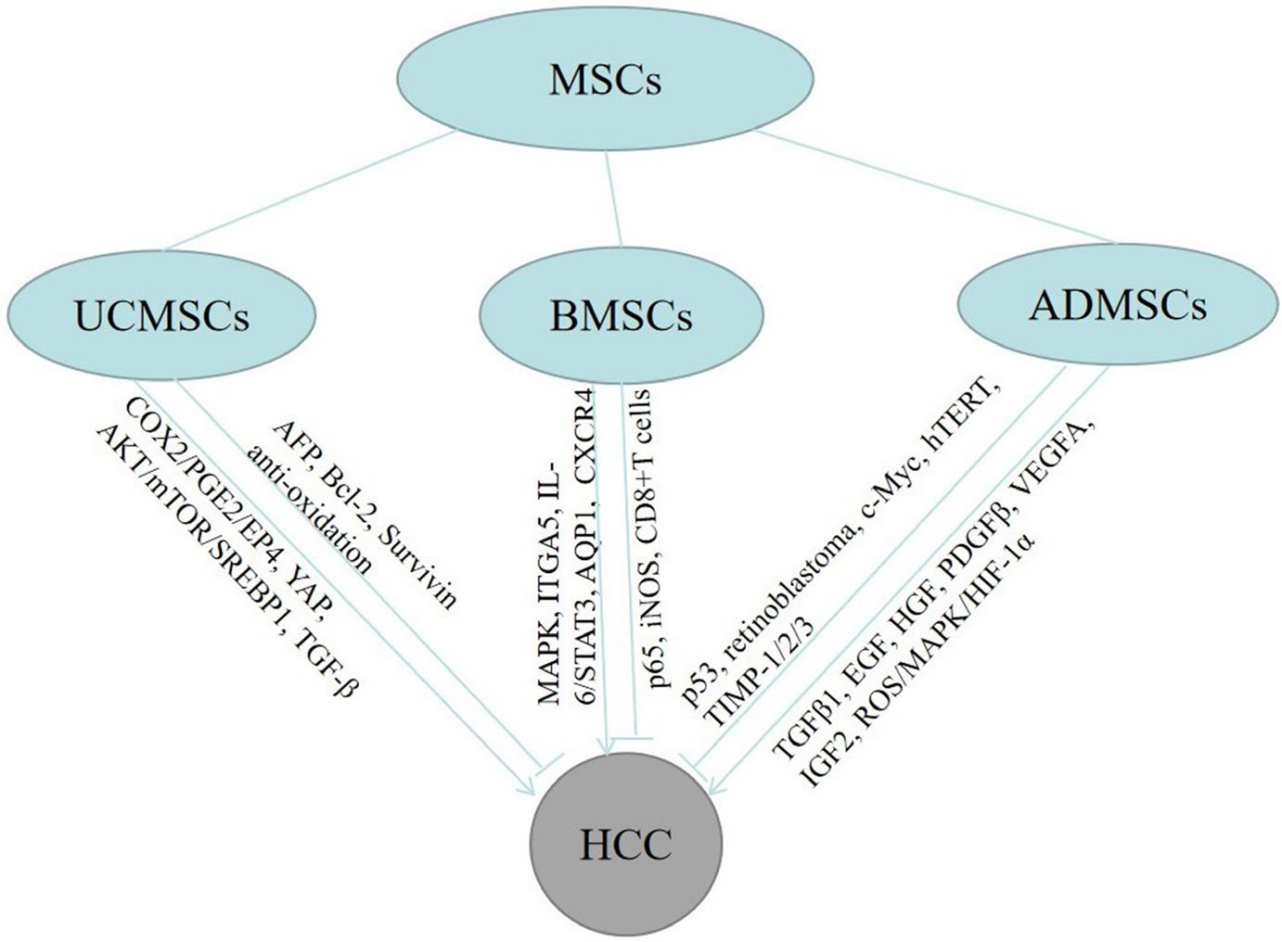
The molecular signaling mechanism of three main mesenchymal stem cells acting on HCC. MSCs, mesenchymal stem cells; UCMSCs, umbilical cord mesenchymal stem cells; BMSCs, bone marrow mesenchymal stem cells; ADMSCs, adipose‐derived mesenchymal stem cells; HCC, hepatocellular carcinoma

**FIGURE 3:**
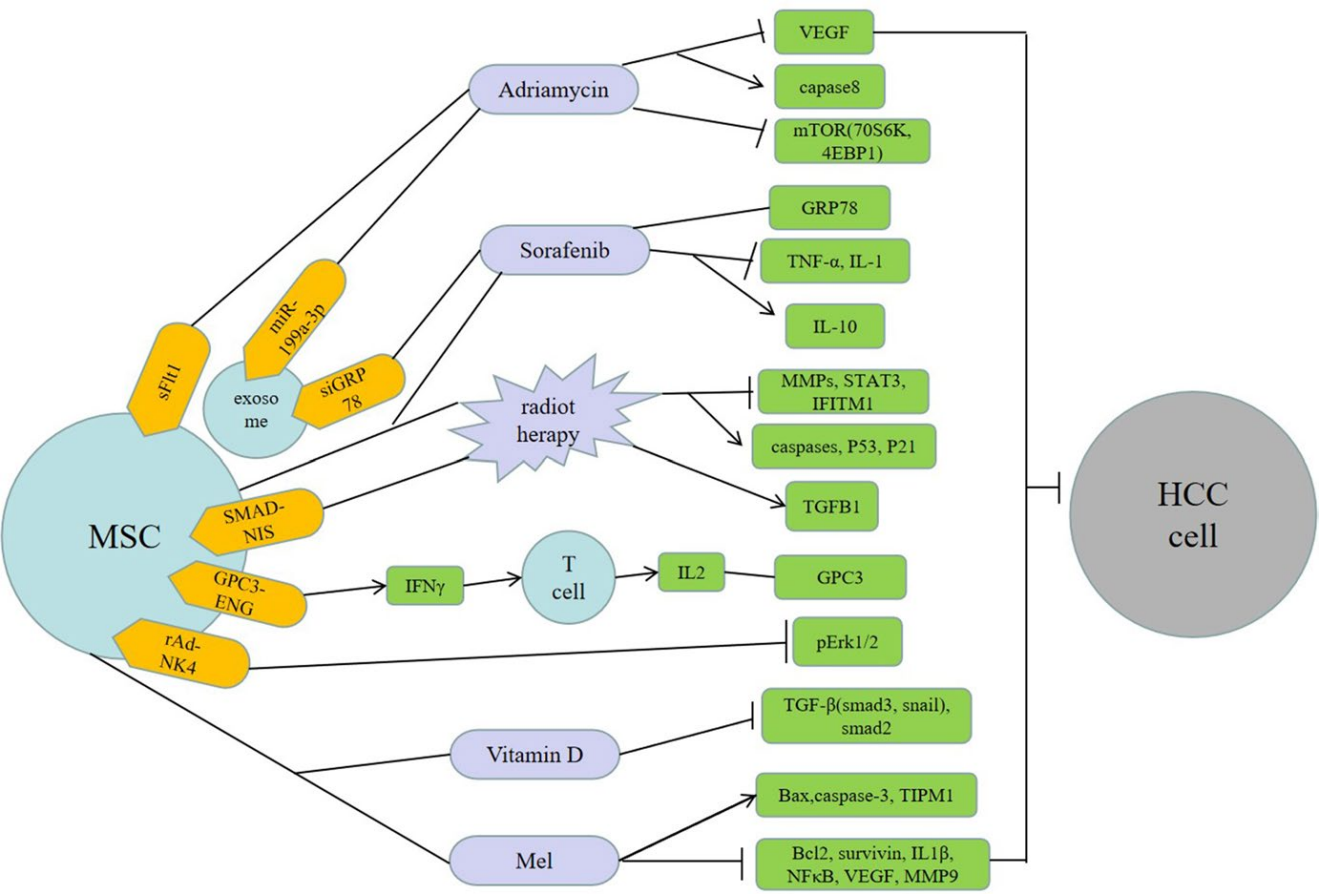
The combination therapies of mesenchymal stem cells (MSCs) for hepatocellular carcinoma (HCC). MSCs or their exosomes can be genetically modified (yellow) or combined with other treatments (purple) to inhibit the proliferation and progression of HCC cells through a variety of molecular signaling mechanisms (green)

MSCs not only can be recruited into the TME to become the HCC cell source, but launch the inhibition or promotion effects on HCC progression through multiple molecular signaling pathways (Figure 2). Yet, in spite of considerable research data highlight the prospect of MSCs and their secreted exosomes be modified or directly used to treat HCC (Figure 3), but the two‐way effect of MSCs on tumor vicious process still should not be ignored. When MSCs are used for treatment, the possibility that they may induce tumor recurrence exists.^101^, ^102^ Moreover, the close relationship between MSCs and LCSCs and their critical roles in drug resistance may lead to continued tumor relapse.^103^, ^104^ Therefore, we should be more cautious in MSCs‐based therapies for HCC and dedicated to revealing the mechanism of the MSCs bidirectional effect on HCC. More data need to be acquired on whether MSCs functions are in correlation with MSCs sources or HCC subtypes and what are the connections and differences between MSCs and TA‐MSCs? Intriguingly, the interaction of MSCs and LCSCs may exert an enormous function on MSCs accelerating the progress of HCC. However, research about it needs further to be done. In terms of treatment, we still need to further disclose the molecular mechanism of MSCs migration to improve recruitment efficiency and the effectiveness of targeted therapy. The interaction between MSCs and infiltrating immune cells also provides a new perspective for future HCC treatment. Foremost, blocking the malignant transformation and tumor promotion of MSCs in HCC targeted therapy is the key to making MSCs an ideal therapy for HCC. We believe that based on the outstanding properties of homing and inhibiting HCC, MSCs or their exosomes may constitute a compelling treatment or adjuvant therapy for HCC in the future.

## CONFLICT OF INTEREST

The authors report no conflict of interest in this work.

## AUTHOR CONTRIBUTIONS

Xiaoli Zhang contributed to literature analysis and manuscript writing. Na Li contributed to literature search. Ying Zhu contributed to supervision and revise. Wei Wen contributed to revise.

## ETHICAL APPROVAL

Ethical approval was not required for this review article.

## Data Availability

The data used to support the findings of this study are available from the corresponding author upon request.

## References

[1] Bray F , Ferlay J , Soerjomataram I , Siegel RL , Torre LA , Jemal A . Global cancer statistics 2018: GLOBOCAN estimates of incidence and mortality worldwide for 36 cancers in 185 countries. CA Cancer J Clin. 2018;68:394‐424. 30207593 10.3322/caac.21492

[2] Petrick JL , Florio AA , Znaor A , et al. International trends in hepatocellular carcinoma incidence, 1978–2012. Int J Cancer. 2020;147:317‐330. 31597196 10.1002/ijc.32723 PMC7470451

[3] Chan LH , Luk ST , Ma S . Turning hepatic cancer stem cells inside out – a deeper understanding through multiple perspectives. Mol Cells. 2015;38:202‐209. 25666349 10.14348/molcells.2015.2356 PMC4363719

[4] Tahmasebi Birgani M , Carloni V . Tumor microenvironment, a paradigm in hepatocellular carcinoma progression and therapy. Int J Mol Sci. 2017;18. 10.3390/ijms18020405 PMC5343939 28216578

[5] Molina ER , Smith BT , Shah SR , Shin H , Mikos AG . Immunomodulatory properties of stem cells and bioactive molecules for tissue engineering. J Control Release. 2015;219:107‐118. 26307349 10.1016/j.jconrel.2015.08.038

[6] Zong C , Zhang H , Yang X , et al. The distinct roles of mesenchymal stem cells in the initial and progressive stage of hepatocarcinoma. Cell Death Dis. 2018;9:345. 29497038 10.1038/s41419-018-0366-7 PMC5832809

[7] McLean K , Gong Y , Choi Y , et al. Human ovarian carcinoma–associated mesenchymal stem cells regulate cancer stem cells and tumorigenesis via altered BMP production. J Clin Invest. 2011;121:3206‐3219. 21737876 10.1172/JCI45273 PMC3148732

[8] Liu S , Ginestier C , Ou SJ , et al. Breast cancer stem cells are regulated by mesenchymal stem cells through cytokine networks. Cancer Res. 2011;71:614‐624. 21224357 10.1158/0008-5472.CAN-10-0538 PMC3100554

[9] Luo J , Ok Lee S , Liang L , et al. Infiltrating bone marrow mesenchymal stem cells increase prostate cancer stem cell population and metastatic ability via secreting cytokines to suppress androgen receptor signaling. Oncogene. 2014;33:2768‐2778. 23792449 10.1038/onc.2013.233

[10] Riggi N , Suvà ML , De Vito C , et al. EWS‐FLI‐1 modulates miRNA145 and SOX2 expression to initiate mesenchymal stem cell reprogramming toward Ewing sarcoma cancer stem cells. Genes Dev. 2010;24:916‐932. 20382729 10.1101/gad.1899710 PMC2861191

[11] Ma X , Liu J , Yang X , et al. Mesenchymal stem cells maintain the stemness of colon cancer stem cells via interleukin‐8/mitogen‐activated protein kinase signaling pathway. Exp Biol Med. 2020;245:562‐575. 10.1177/1535370220910690 PMC7158600 32122165

[12] Chen J , Ji T , Wu DI , et al. Human mesenchymal stem cells promote tumor growth via MAPK pathway and metastasis by epithelial mesenchymal transition and integrin α5 in hepatocellular carcinoma. Cell Death Dis. 2019;10:425. 31142737 10.1038/s41419-019-1622-1 PMC6541606

[13] Wang C , Hu J , Chen Z , et al. Reversibility of hAT‐MSCs phenotypic and metabolic changes after exposure to and withdrawal from HCC‐conditioned medium through regulation of the ROS/MAPK/HIF‐1α signaling pathway. Stem Cell Res Ther. 2020;11:506. 33246501 10.1186/s13287-020-02010-0 PMC7694319

[14] Liu Y , Ren H , Zhou Y , et al. The hypoxia conditioned mesenchymal stem cells promote hepatocellular carcinoma progression through YAP mediated lipogenesis reprogramming. J Exp Clin Cancer Res. 2019;38:228. 31142342 10.1186/s13046-019-1219-7 PMC6540399

[15] Teshima T , Matsumoto H , Koyama H . Soluble factors from adipose tissue‐derived mesenchymal stem cells promote canine hepatocellular carcinoma cell proliferation and invasion. PLoS One. 2018;13:e0191539. 29346427 10.1371/journal.pone.0191539 PMC5773216

[16] Yan X , Zhang D , Wu W , et al. Mesenchymal stem cells promote hepatocarcinogenesis via lncRNA‐MUF interaction with ANXA2 and miR‐34a. Cancer Res. 2017;77:6704‐6716. 28947421 10.1158/0008-5472.CAN-17-1915

[17] Mi F , Gong L . Secretion of interleukin‐6 by bone marrow mesenchymal stem cells promotes metastasis in hepatocellular carcinoma. Biosci Rep. 2017;37. 10.1042/BSR20170181 PMC5567045 28659496

[18] Pelagalli A , Nardelli A , Fontanella R , Zannetti A . Inhibition of AQP1 hampers osteosarcoma and hepatocellular carcinoma progression mediated by bone marrow‐derived mesenchymal stem cells. Int J Mol Sci. 2016;17. 10.3390/ijms17071102 PMC4964478 27409610

[19] Fontanella R , Pelagalli A , Nardelli A , et al. A novel antagonist of CXCR4 prevents bone marrow‐derived mesenchymal stem cell‐mediated osteosarcoma and hepatocellular carcinoma cell migration and invasion. Cancer Lett. 2016;370:100‐107. 26517945 10.1016/j.canlet.2015.10.018

[20] Liu C , Liu Y , Xu XX , Guo X , Sun GW , Ma XJ . Mesenchymal stem cells enhance the metastasis of 3D‐cultured hepatocellular carcinoma cells. BMC Cancer. 2016;16:566. 27475525 10.1186/s12885-016-2595-4 PMC4967520

[21] Liu J , Shi Y , Han J , Zhang Y , Cao Z , Cheng J . Quantitative tracking tumor suppression efficiency of human umbilical cord‐derived mesenchymal stem cells by bioluminescence imaging in mice hepatoma model. Int J Stem Cells. 2020;13:104‐115. 31887848 10.15283/ijsc19098 PMC7119203

[22] Alshareeda AT , Alsowayan B , Almubarak A , Alghuwainem A , Alshawakir Y , Alahmed M . Exploring the potential of mesenchymal stem cell sheet on the development of hepatocellular carcinoma in vivo. J Vis Exp. 2018;(139):57805. 10.3791/57805 PMC6235166 30272642

[23] Jiang W , Tan Y , Cai M , et al. Human umbilical cord MSC‐derived exosomes suppress the development of CCl(4)‐induced liver injury through antioxidant effect. Stem Cells Int. 2018;2018:6079642. 29686713 10.1155/2018/6079642 PMC5857330

[24] Tang Y‐M , Bao W‐M , Yang J‐H , et al. Umbilical cord‐derived mesenchymal stem cells inhibit growth and promote apoptosis of HepG2 cells. Mol Med Rep. 2016;14:2717‐2724. 27485485 10.3892/mmr.2016.5537

[25] Liu Q‐W , Li J‐Y , Zhang X‐C , et al. Human amniotic mesenchymal stem cells inhibit hepatocellular carcinoma in tumour‐bearing mice. J Cell Mol Med. 2020;24:10525‐10541. 32798252 10.1111/jcmm.15668 PMC7521292

[26] Wu Y , Chen X , Zhao Y , Wang Y , Li Y , Xiang C . Genome‐wide DNA methylation and hydroxymethylation analysis reveal human menstrual blood‐derived stem cells inhibit hepatocellular carcinoma growth through oncogenic pathway suppression via regulating 5‐hmC in enhancer elements. Stem Cell Res Ther. 2019;10:151. 31151404 10.1186/s13287-019-1243-8 PMC6544940

[27] Serhal R , Saliba N , Hilal G , et al. Effect of adipose‐derived mesenchymal stem cells on hepatocellular carcinoma: in vitro inhibition of carcinogenesis. World J Gastroenterol. 2019;25:567‐583. 30774272 10.3748/wjg.v25.i5.567 PMC6371009

[28] Ye F , Jiang J , Zong C , et al. Sirt1‐overexpressing mesenchymal stem cells drive the anti‐tumor effect through their pro‐inflammatory capacity. Mol Ther. 2020;28:874‐888. 32027844 10.1016/j.ymthe.2020.01.018 PMC7054827

[29] Hajighasemlou S , Nikbakht M , Pakzad S , et al. Sorafenib and mesenchymal stem cell therapy: a promising approach for treatment of HCC. Evid Based Complement Alternat Med. 2020;2020:9602728. 32617114 10.1155/2020/9602728 PMC7312705

[30] Seyhoun I , Hajighasemlou S , Muhammadnejad S , et al. Combination therapy of sorafenib with mesenchymal stem cells as a novel cancer treatment regimen in xenograft models of hepatocellular carcinoma. J Cell Physiol. 2019;234:9495‐9503. 30362607 10.1002/jcp.27637

[31] Niu J , Wang Y , Wang J , Bin L , Hu X . Delivery of sFIT‐1 engineered MSCs in combination with a continuous low‐dose doxorubicin treatment prevents growth of liver cancer. Aging. 2016;8:3520‐3534. 28039440 10.18632/aging.101146 PMC5270684

[32] Li G , Miao F , Zhu J , Chen Y . Anti‐angiogenesis gene therapy for hepatocellular carcinoma via systemic injection of mesenchymal stem cells engineered to secrete soluble Flt‐1. Mol Med Rep. 2017;16:5799‐5806. 28849176 10.3892/mmr.2017.7310 PMC5865760

[33] Seyhoun I , Hajighasemlou S , Ai J , et al. Novel combination of mesenchymal stem cell‐conditioned medium with sorafenib have synergistic antitumor effect of hepatocellular carcinoma cells. Asian Pac J Cancer Prev. 2019;20:263‐267. 30678447 10.31557/APJCP.2019.20.1.263 PMC6485565

[34] Lou G , Chen L , Xia C , et al. MiR‐199a‐modified exosomes from adipose tissue‐derived mesenchymal stem cells improve hepatocellular carcinoma chemosensitivity through mTOR pathway. J Exp Clin Cancer Res. 2020;39:4. 31898515 10.1186/s13046-019-1512-5 PMC6941283

[35] Li H , Yang C , Shi Y , Zhao L . Exosomes derived from siRNA against GRP78 modified bone‐marrow‐derived mesenchymal stem cells suppress Sorafenib resistance in hepatocellular carcinoma. J Nanobiotechnol. 2018;16:103. 10.1186/s12951-018-0429-z PMC6300915 30572882

[36] Wu L , Tang Q , Yin X , et al. The therapeutic potential of adipose tissue‐derived mesenchymal stem cells to enhance radiotherapy effects on hepatocellular carcinoma. Front Cell Dev Biol. 2019;7:267. 31781559 10.3389/fcell.2019.00267 PMC6861425

[37] Schug C , Urnauer S , Jaeckel C , et al. TGFB1‐driven mesenchymal stem cell‐mediated NIS gene transfer. Endocr Relat Cancer. 2019;26:89‐101. 30121623 10.1530/ERC-18-0173

[38] Schug C , Kitzberger C , Sievert W , et al. Radiation‐induced amplification of TGFB1‐induced mesenchymal stem cell‐mediated sodium iodide symporter (NIS) gene (131)I therapy. Clin Cancer Res. 2019;25:5997‐6008. 31196853 10.1158/1078-0432.CCR-18-4092

[39] Yoon A‐R , Hong J , Li Y , et al. Mesenchymal stem cell‐mediated delivery of an oncolytic adenovirus enhances antitumor efficacy in hepatocellular carcinoma. Cancer Res. 2019;79:4503‐4514. 31289131 10.1158/0008-5472.CAN-18-3900

[40] Zhang J , Hou L , Wu X , et al. Inhibitory effect of genetically engineered mesenchymal stem cells with Apoptin on hepatoma cells in vitro and in vivo. Mol Cell Biochem. 2016;416:193‐203. 27142531 10.1007/s11010-016-2707-0

[41] El‐Magd MA , Mohamed Y , El‐Shetry ES , et al. Melatonin maximizes the therapeutic potential of non‐preconditioned MSCs in a DEN‐induced rat model of HCC. Biomed Pharmacother. 2019;114:108732. 10.1016/j.biopha.2019.108732 30925457

[42] Saad El‐Din S , Fouad H , Rashed LA , Mahfouz S , Hussein RE . Impact of mesenchymal stem cells and vitamin D on transforming growth factor beta signaling pathway in hepatocellular carcinoma in rats. Asian Pac J Cancer Prev. 2018;19:905‐912. 29693337 10.22034/APJCP.2018.19.4.905 PMC6031804

[43] Szoor A , Vaidya A , Velasquez MP , et al. T cell‐activating mesenchymal stem cells as a biotherapeutic for HCC. Mol Ther Oncolytics. 2017;6:69‐79. 28856237 10.1016/j.omto.2017.07.002 PMC5562179

[44] Cai C , Hou L , Zhang J , et al. The inhibitory effect of mesenchymal stem cells with rAd‐NK4 on liver cancer. Appl Biochem Biotechnol. 2017;183:444‐459. 28353041 10.1007/s12010-017-2456-x

[45] Cao H , Xu W , Qian H , et al. Mesenchymal stem cell‐like cells derived from human gastric cancer tissues. Cancer Lett. 2009;274(1):61‐71. 18849111 10.1016/j.canlet.2008.08.036

[46] Yan X‐L , Fu C‐J , Chen L , et al. Mesenchymal stem cells from primary breast cancer tissue promote cancer proliferation and enhance mammosphere formation partially via EGF/EGFR/Akt pathway. Breast Cancer Res Treat. 2012;132(1):153‐164. 21584665 10.1007/s10549-011-1577-0

[47] Lin J‐T , Wang J‐Y , Chen M‐K , et al. Colon cancer mesenchymal stem cells modulate the tumorigenicity of colon cancer through interleukin 6. Exp Cell Res. 2013;319(14):2216‐2229. 23751564 10.1016/j.yexcr.2013.06.003

[48] Waghray M , Yalamanchili M , Dziubinski M , et al. GM‐CSF mediates mesenchymal‐epithelial cross‐talk in pancreatic cancer. Cancer Discov. 2016;6(8):886‐899. 27184426 10.1158/2159-8290.CD-15-0947 PMC5549011

[49] Hassanzadeh A , Rahman HS , Markov A , et al. Mesenchymal stem/stromal cell‐derived exosomes in regenerative medicine and cancer; overview of development, challenges, and opportunities. Stem Cell Res Ther. 2021;12(1):297. 34020704 10.1186/s13287-021-02378-7 PMC8138094

[50] Bayo J , Real A , Fiore EJ , et al. IL‐8, GRO and MCP‐1 produced by hepatocellular carcinoma microenvironment determine the migratory capacity of human bone marrow‐derived mesenchymal stromal cells without affecting tumor aggressiveness. Oncotarget. 2017;8(46):80235‐80248. 29113298 10.18632/oncotarget.10288 PMC5655193

[51] Gao Y , Zhou Z , Lu S , et al. Chemokine CCL15 mediates migration of human bone marrow‐derived mesenchymal stem cells toward hepatocellular carcinoma. Stem Cells. 2016;34(4):1112‐1122. 26763650 10.1002/stem.2275

[52] Lejmi E , Perriraz N , Clément S , et al. Inflammatory chemokines MIP‐1δ and MIP‐3α are involved in the migration of multipotent mesenchymal stromal cells induced by hepatoma cells. Stem Cells Dev. 2015;24(10):1223‐1235. 25579056 10.1089/scd.2014.0176 PMC4425419

[53] Mardomi A , Sabzichi M , Hussein Somi M , et al. Trafficking mechanism of bone marrow‐derived mesenchymal stem cells toward hepatocellular carcinoma HepG2 cells by modulating Endoglin, CXCR4 and TGF‐β. Cell Mol Biol (Noisy‐le‐grand). 2016;62:81‐86. 27755957

[54] Schmohl KA , Müller AM , Wechselberger A , et al. Thyroid hormones and tetrac: new regulators of tumour stroma formation via integrin αvβ3. Endocr Relat Cancer. 2015;22(6):941‐952. 26307023 10.1530/ERC-15-0245

[55] Xie C , Yang Z , Suo Y , et al. Systemically infused mesenchymal stem cells show different homing profiles in healthy and tumor mouse models. Stem Cells Transl Med. 2017;6(4):1120‐1131. 28205428 10.1002/sctm.16-0204 PMC5442841

[56] Rosová I , Dao M , Capoccia B , Link D , Nolta JA . Hypoxic preconditioning results in increased motility and improved therapeutic potential of human mesenchymal stem cells. Stem Cells. 2008;26(8):2173‐2182. 18511601 10.1634/stemcells.2007-1104 PMC3017477

[57] Tutter M , Schug C , Schmohl KA , et al. Regional hyperthermia enhances mesenchymal stem cell recruitment to tumor stroma: implications for mesenchymal stem cell‐based tumor therapy. Mol Ther. 2021;29(2):788‐803. 33068779 10.1016/j.ymthe.2020.10.009 PMC7854278

[58] Batten P , Sarathchandra P , Antoniw JW , et al. Human mesenchymal stem cells induce T cell anergy and downregulate T cell allo‐responses via the TH2 pathway: relevance to tissue engineering human heart valves. Tissue Eng. 2006;12(8):2263‐2273. 16968166 10.1089/ten.2006.12.2263

[59] Sato K , Ozaki K , Oh I , et al. Nitric oxide plays a critical role in suppression of T‐cell proliferation by mesenchymal stem cells. Blood. 2007;109(1):228‐234. 16985180 10.1182/blood-2006-02-002246

[60] Gao W‐X , Sun Y‐Q , Shi J , et al. Effects of mesenchymal stem cells from human induced pluripotent stem cells on differentiation, maturation, and function of dendritic cells. Stem Cell Res Ther. 2017;8(1):48. 28253916 10.1186/s13287-017-0499-0 PMC5333407

[61] Mathew E , Brannon AL , Del Vecchio A , et al. Mesenchymal stem cells promote pancreatic tumor growth by inducing alternative polarization of macrophages. Neoplasia. 2016;18(3):142‐151. 26992915 10.1016/j.neo.2016.01.005 PMC4796803

[62] Zhang Q‐Z , Su W‐R , Shi S‐H , et al. Human gingiva‐derived mesenchymal stem cells elicit polarization of m2 macrophages and enhance cutaneous wound healing. Stem Cells. 2010;28(10):1856‐1868. 20734355 10.1002/stem.503 PMC3114043

[63] Selleri S , Bifsha P , Civini S , et al. Human mesenchymal stromal cell‐secreted lactate induces M2‐macrophage differentiation by metabolic reprogramming. Oncotarget. 2016;7(21):30193‐30210. 27070086 10.18632/oncotarget.8623 PMC5058674

[64] Han Z , Tian Z , Lv G , et al. Immunosuppressive effect of bone marrow‐derived mesenchymal stem cells in inflammatory microenvironment favours the growth of B16 melanoma cells. J Cell Mol Med. 2011;15(11):2343‐2352. 21091630 10.1111/j.1582-4934.2010.01215.x PMC3822946

[65] Djouad F , Plence P , Bony C , et al. Immunosuppressive effect of mesenchymal stem cells favors tumor growth in allogeneic animals. Blood. 2003;102(10):3837‐3844. 12881305 10.1182/blood-2003-04-1193

[66] Sotiropoulou PA , Perez SA , Gritzapis AD , Baxevanis CN , Papamichail M . Interactions between human mesenchymal stem cells and natural killer cells. Stem Cells. 2006;24(1):74‐85. 16099998 10.1634/stemcells.2004-0359

[67] Di Nicola M , Carlo‐Stella C , Magni M , et al. Human bone marrow stromal cells suppress T‐lymphocyte proliferation induced by cellular or nonspecific mitogenic stimuli. Blood. 2002;99(10):3838‐3843. 11986244 10.1182/blood.v99.10.3838

[68] Akiyama K , Chen C , Wang D , et al. Mesenchymal‐stem‐cell‐induced immunoregulation involves FAS‐ligand‐/FAS‐mediated T cell apoptosis. Cell Stem Cell. 2012;10(5):544‐555. 22542159 10.1016/j.stem.2012.03.007 PMC3348385

[69] O'Connor BP , Vogel LA , Zhang W , et al. Imprinting the fate of antigen‐reactive B cells through the affinity of the B cell receptor. J Immunol. 2006;177(11):7723‐7732. 17114443 10.4049/jimmunol.177.11.7723 PMC2819292

[70] Barcellos‐de‐Souza P , Comito G , Pons‐Segura C , et al. Mesenchymal stem cells are recruited and activated into carcinoma‐associated fibroblasts by prostate cancer microenvironment‐derived TGF‐β1. Stem Cells. 2016;34(10):2536‐2547. 27300750 10.1002/stem.2412

[71] Shangguan L , Ti X , Krause U , et al. Inhibition of TGF‐β/Smad signaling by BAMBI blocks differentiation of human mesenchymal stem cells to carcinoma‐associated fibroblasts and abolishes their protumor effects. Stem Cells. 2012;30(12):2810‐2819. 23034983 10.1002/stem.1251

[72] Ji J , Eggert T , Budhu A , et al. Hepatic stellate cell and monocyte interaction contributes to poor prognosis in hepatocellular carcinoma. Hepatology (Baltimore, MD). 2015;62(2):481‐495. 10.1002/hep.27822 PMC4515211 25833323

[73] Zhao W , Su W , Kuang P , et al. The role of hepatic stellate cells in the regulation of T‐cell function and the promotion of hepatocellular carcinoma. Int J Oncol. 2012;41(2):457‐464. 22641338 10.3892/ijo.2012.1497 PMC3582803

[74] Zhao W , Zhang L , Yin Z , et al. Activated hepatic stellate cells promote hepatocellular carcinoma development in immunocompetent mice. Int J Cancer. 2011;129(11):2651‐2661. 21213212 10.1002/ijc.25920

[75] Cheng Y , Li H , Deng Y , et al. Cancer‐associated fibroblasts induce PDL1^+^ neutrophils through the IL6‐STAT3 pathway that foster immune suppression in hepatocellular carcinoma. Cell Death Dis. 2018;9(4):422. 29556041 10.1038/s41419-018-0458-4 PMC5859264

[76] Ohlsson LB , Varas L , Kjellman C , Edvardsen K , Lindvall M . Mesenchymal progenitor cell‐mediated inhibition of tumor growth in vivo and in vitro in gelatin matrix. Exp Mol Pathol. 2003;75(3):248‐255. 14611816 10.1016/j.yexmp.2003.06.001

[77] Cassatella MA , Mosna F , Micheletti A , et al. Toll‐like receptor‐3‐activated human mesenchymal stromal cells significantly prolong the survival and function of neutrophils. Stem Cells. 2011;29(6):1001‐1011. 21563279 10.1002/stem.651

[78] Stagg J , Pommey S , Eliopoulos N , Galipeau J . Interferon‐gamma‐stimulated marrow stromal cells: a new type of nonhematopoietic antigen‐presenting cell. Blood. 2006;107(6):2570‐2577. 16293599 10.1182/blood-2005-07-2793

[79] Zheng H , Zou W , Shen J , et al. Opposite effects of coinjection and distant injection of mesenchymal stem cells on breast tumor cell growth. Stem Cells Transl Med. 2016;5(9):1216‐1228. 27352928 10.5966/sctm.2015-0300 PMC4996440

[80] Qian H , Ding X , Zhang J , et al. Cancer stemness and metastatic potential of the novel tumor cell line K3: an inner mutated cell of bone marrow‐derived mesenchymal stem cells. Oncotarget. 2017;8:39522‐39533. 28465472 10.18632/oncotarget.17133 PMC5503629

[81] Xu X , Qian H , Zhu W , et al. Isolation of cancer stem cells from transformed human mesenchymal stem cell line F6. J Mol Med (Berl). 2010;88:1181‐1190. 20697686 10.1007/s00109-010-0659-5

[82] Maj M , Kokocha A , Bajek A , Drewa T . The effects of adipose‐derived stem cells on CD133‐expressing bladder cancer cells. J Cell Biochem. 2019;120:11562‐11572. 10.1002/jcb.28436 30746788

[83] Vulcano F , Milazzo L , Ciccarelli C , et al. Wharton's jelly mesenchymal stromal cells have contrasting effects on proliferation and phenotype of cancer stem cells from different subtypes of lung cancer. Exp Cell Res. 2016;345:190‐198. 27343631 10.1016/j.yexcr.2016.06.003

[84] El‐Badawy A , Ghoneim MA , Gabr MM , et al. Cancer cell‐soluble factors reprogram mesenchymal stromal cells to slow cycling, chemoresistant cells with a more stem‐like state. Stem Cell Res Ther. 2017;8:254. 29115987 10.1186/s13287-017-0709-9 PMC5688803

[85] Eun JR , Jung YJ , Zhang Y , et al. Hepatoma SK Hep‐1 cells exhibit characteristics of oncogenic mesenchymal stem cells with highly metastatic capacity. PLoS One. 2014;9:e110744. 25338121 10.1371/journal.pone.0110744 PMC4206444

[86] Wu M , Lin Z , Li X , et al. HULC cooperates with MALAT1 to aggravate liver cancer stem cells growth through telomere repeat‐binding factor 2. Sci Rep. 2016;6:36045. 27782152 10.1038/srep36045 PMC5080550

[87] Li X , Wang J , Pan Y , et al. Long non‐coding RNA HULC affects the proliferation, apoptosis, migration, and invasion of mesenchymal stem cells. Exp Biol Med. 2018;243:1074‐1082. 10.1177/1535370218804781 PMC6434460 30269516

[88] Li X , Song Y , Liu F , et al. Long non‐coding RNA MALAT1 promotes proliferation, angiogenesis, and immunosuppressive properties of mesenchymal stem cells by inducing VEGF and IDO. J Cell Biochem. 2017;118:2780‐2791. 28176360 10.1002/jcb.25927

[89] Hou J , Zhao N , Zhu P , Chang J , Du Y , Shen W . Irradiated mesenchymal stem cells support stemness maintenance of hepatocellular carcinoma stem cells through Wnt/β‐catenin signaling pathway. Cell Biosci. 2020;10:93. 32774840 10.1186/s13578-020-00449-5 PMC7398068

[90] Hu Y , Zai H , Jiang W , Yao Y , Ou Z , Zhu Q . miR‐126 in extracellular vesicles derived from hepatoblastoma cells promotes the tumorigenesis of hepatoblastoma through inducing the differentiation of BMSCs into cancer stem cells. J Immunol Res. 2021;2021:6744715. 34746322 10.1155/2021/6744715 PMC8570887

[91] Vilchez V , Turcios L , Marti F , Gedaly R . Targeting Wnt/β‐catenin pathway in hepatocellular carcinoma treatment. World J Gastroenterol. 2016;22:823‐832. 26811628 10.3748/wjg.v22.i2.823 PMC4716080

[92] Wang S , Xu M , Li X , et al. Exosomes released by hepatocarcinoma cells endow adipocytes with tumor‐promoting properties. J Hematol Oncol. 2018;11:82. 29898759 10.1186/s13045-018-0625-1 PMC6001126

[93] Qiao L , Zhao TJ , Wang FZ , Shan CL , Ye LH , Zhang XD . NF‐kappaB downregulation may be involved the depression of tumor cell proliferation mediated by human mesenchymal stem cells. Acta Pharmacol Sin. 2008;29:333‐340. 18298898 10.1111/j.1745-7254.2008.00751.x

[94] Abdel Aziz MT , Khaled HM , El Hindawi A , et al. Effect of mesenchymal stem cells and a novel curcumin derivative on Notch1 signaling in hepatoma cell line. Biomed Res Int. 2013;2013:129629. 10.1155/2013/129629 PMC3760179 24024180

[95] Zhao W , Ren G , Zhang L , et al. Efficacy of mesenchymal stem cells derived from human adipose tissue in inhibition of hepatocellular carcinoma cells in vitro. Cancer Biother Radiopharm. 2012;27:606‐613. 22917212 10.1089/cbr.2011.1150

[96] Li G‐C , Ye Q‐H , Xue Y‐H , et al. Human mesenchymal stem cells inhibit metastasis of a hepatocellular carcinoma model using the MHCC97‐H cell line. Cancer Sci. 2010;101:2546‐2553. 20942864 10.1111/j.1349-7006.2010.01738.x PMC11159711

[97] Qiao L , Xu ZL , Zhao TJ , Ye LH , Zhang XD . Dkk‐1 secreted by mesenchymal stem cells inhibits growth of breast cancer cells via depression of Wnt signalling. Cancer Lett. 2008;269:67‐77. 18571836 10.1016/j.canlet.2008.04.032

[98] Hou L , Wang X , Zhou Y , et al. Inhibitory effect and mechanism of mesenchymal stem cells on liver cancer cells. Tumour Biol. 2014;35:1239‐1250. 24136741 10.1007/s13277-013-1165-5

[99] Endaya B , Guan SP , Newman JP , et al. Human mesenchymal stem cells preferentially migrate toward highly oncogenic human hepatocellular carcinoma cells with activated EpCAM signaling. Oncotarget. 2017;8:54629‐54639. 28903370 10.18632/oncotarget.17633 PMC5589609

[100] Alzahrani FA , El‐Magd MA , Abdelfattah‐Hassan A , et al. Potential effect of exosomes derived from cancer stem cells and MSCs on progression of DEN‐induced HCC in rats. Stem Cells Int. 2018;2018:8058979. 30224923 10.1155/2018/8058979 PMC6129855

[101] Chaput B , Foucras L , Le Guellec S , Grolleau JL , Garrido I . Recurrence of an invasive ductal breast carcinoma 4 months after autologous fat grafting. Plast Reconstr Surg. 2013;131:123e‐124e. 10.1097/PRS.0b013e318272a1f6 23271541

[102] Perrot P , Rousseau J , Bouffaut A‐L , et al. Safety concern between autologous fat graft, mesenchymal stem cell and osteosarcoma recurrence. PLoS One. 2010;5:e10999. 20544017 10.1371/journal.pone.0010999 PMC2882323

[103] Coffman LG , Choi YJ , McLean K , Allen BL , di Magliano MP , Buckanovich RJ . Human carcinoma‐associated mesenchymal stem cells promote ovarian cancer chemotherapy resistance via a BMP4/HH signaling loop. Oncotarget. 2016;7:6916‐6932. 26755648 10.18632/oncotarget.6870 PMC4872758

[104] Daverey A , Drain AP , Kidambi S . Physical intimacy of breast cancer cells with mesenchymal stem cells elicits trastuzumab resistance through Src activation. Sci Rep. 2015;5:13744. 26345302 10.1038/srep13744 PMC4561910

